# 

**Published:** 1877-03

**Authors:** 


CONTENTS.

| | PAGE | | PAGE |
|---|---|---|---|
| LIFE INSURANCE | 61 | THINK WELL, RATHER THAN EVIL | 75 |
| ANNUAL AILMENTS | 64 | SEARCH FOR WIVES | 76 |
| THE THREE P'S | 68 | SPRAINS | 77 |
| COFFEE DRINKING | 69 | HEART AFFECTIONS | 78 |
| PURE AIR IN MEDICINE | 70 | DIGESTION | 79 |
| STRIKING SENTIMENTS | 71 | LEAD POISON | 80 |
| HUMAN HOPE | 73 | NIGHT AIR | 81 |
| THE LITERATURE OF THE TIMES | 74 | THE LANGUAGE OF FLOWERS | 84 |

